# Supplementary material for: Chronic Neuronal Hyperexcitation Exacerbates Tau Propagation in a Mouse Model of Tauopathy
Source: Int J Mol Sci. 2024 Aug 19;25(16):9004. doi: 10.3390/ijms25169004 (PMC11354494; doi:10.3390/ijms25169004)
Supplement: Supplementary file 1 [file ijms-25-09004-s001.zip › ijms-3137308-supplementary.pdf]

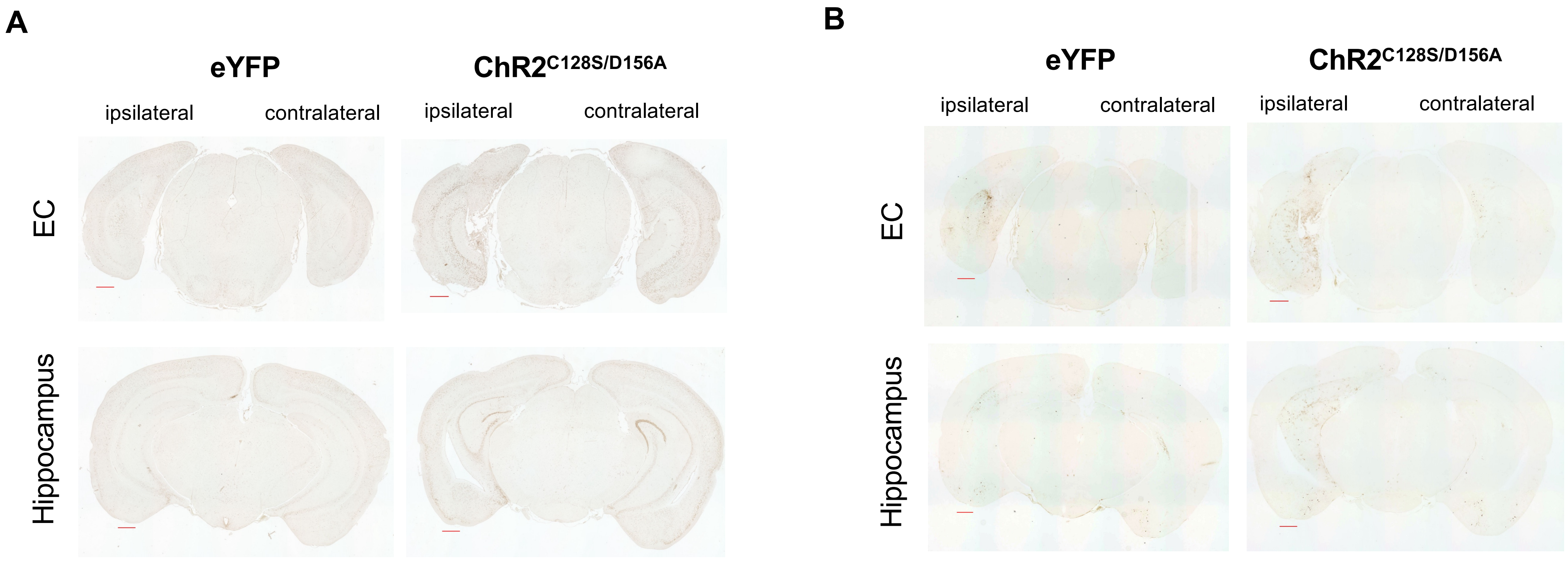

Figure S1. Representative low-magnification images of c-fos (A) and PHF-1 (B) staining in EC and hippocampus following injection of AAV and tau fibrils in PS19 mice. Scale bars, 20 μm.

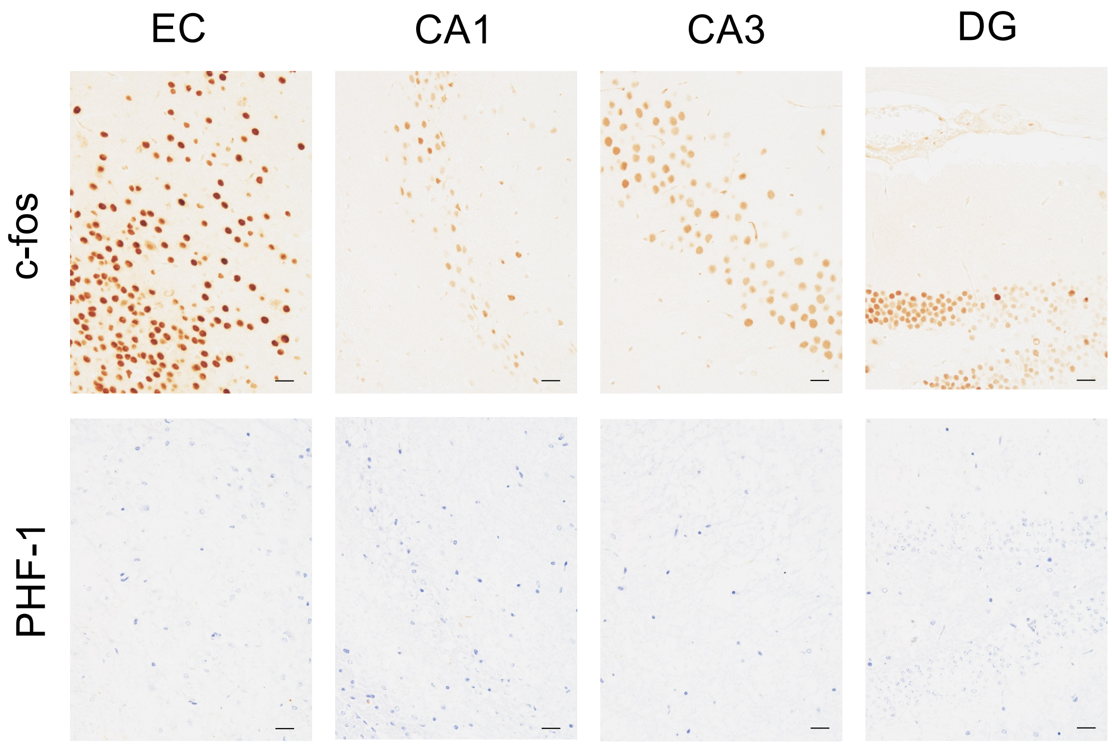

Figure S2. Representative images of c-fos and PHF-1 staining (counterstained with hematoxylin) in EC, CA1, CA3 and DG of hippocampus subjected to 4-week chronic stimulation without injection of tau fibrils in PS19 mice. Scale bars, 20 μm.

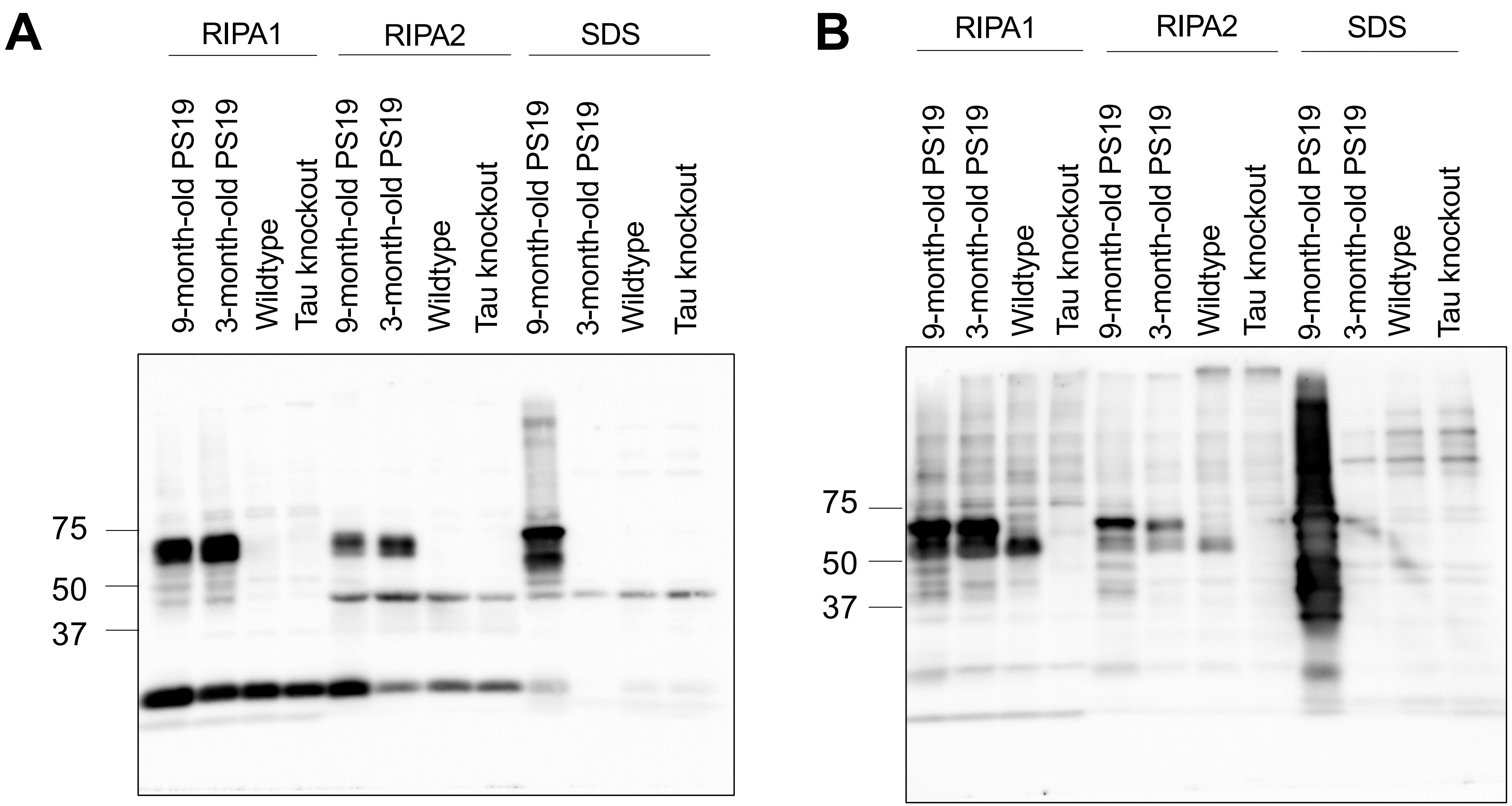

Figure S3. Immunoblots probing for human tau (HJ8.5)(A) and p-tau (PHF-1)(B) in RIPA1, RIPA2 and SDS fractions of PS19, wildtype and tau knockout mouse without injection of tau fibrils.
